# Fluorescent Biocompatible Platinum-Porphyrin–Doped Polymeric Hybrid Particles for Oxygen and Glucose Biosensing

**DOI:** 10.1038/s41598-019-41326-7

**Published:** 2019-03-22

**Authors:** Gaurav Pandey, Rashmi Chaudhari, Bhavana Joshi, Sandeep Choudhary, Jaspreet Kaur, Abhijeet Joshi

**Affiliations:** 1 Discipline of Biosciences and Biomedical Engineering, Indian Institute of Technology Indore, Khandwa Road, Indore, 453552 India; 2 Department of Biosciences and Bioengineering, Indian Institute of Technology Bombay, Powai, Mumbai 40076 India

## Abstract

Near infrared (NIR) fluorophores like Pt-porphyrin along with analyte specific enzymes require co-encapsulation in biocompatible and biodegradable carriers in order to be transformed into implantable biosensors for efficient and continuous monitoring of analytes in patients. The main objective of this research is to develop natural, biodegradable, biocompatible and a novel co-encapsulated system of Pt-porphyrin encapsulated polymeric nanoparticle and nano-micro hybrid carriers. A sequential emulsification-solvent evaporation and an air driven atomization technique was used for developing above matrices and testing them for fluorescence based oxygen and glucose biosensing. The results indicate Pt-porphyrin can be efficiently encapsulated in Poly-lactic acid (PLA) nanoparticles and PLA-alginate nano-micro particles with sizes ~450 nm and 10 µm, respectively. Biosensing studies have showed a linear fluorescent response in oxygen concentrations ranging from of 0–6 mM (R^2^ = 0.992). The Oxygen sensitivity was transformed into a linear response of glucose catalytic reaction in the range of 0–10 mM (R^2^ = 0.968) with a response time of 4 minutes and a stability over 15 days. We believe that the investigated NIR fluorophores like Pt-Porphyrin based nano/nano-micro hybrid carrier systems are novel means of developing biocompatible biodegradable carriers for developing implantable glucose biosensors which can efficiently manage glucose levels in diabetes.

## Introduction

Diabetes has remained the most common disorder of carbohydrate metabolism globally^1^. Optical and electrochemical detection methods for detection of diabetes by glucose monitoring have remained the mainstay of monitoring and reducing diabetic complications. Commercially available glucometers are commonly used tools working based on amperometric measurement glucose in blood samples. Glucose oxidase (GOx) catalyzed conversion to gluconic acid is utilized as the main mechanism for developing such biosensors. The costs of glucometers are high and typically have a coding system with their strip packing. Recent developments however have excluded the coding system, but the accuracy (±20% error range) of these glucometers is a real concern for most health care practitioners^2^. Glucometers typically involve intermittent (single or multiple) measurement of glucose by withdrawing blood samples and can suffer with inaccuracies creating errors in decision making for dosage of antidiabetic drugs. An ideal glucose biosensor should have properties like continuous, real-time measurement, accurate, non-invasive, stable and without any requirement of calibration of sensor. Accurate dosage decisions can be made using such continuously measuring systems to effectively manage this chronic disorder.

Continuously measuring systems started gaining more attention in order to achieve closeness to ideal biosensors. These systems are portable devices which can measure glucose concentration for many days frequently. Such sensors have components like needle, transmitter and portable device measuring the transmitted signal. However, Sparacino *et al*. described concerns regarding quality of data from continuous glucose monitoring systems and have remained only for Supplementary use^2^. It is believed that continuous glucose monitoring devices can be coupled to insulin pumps to allow for self-management. Limitations such as lag time, biofouling, inflammation, fibrous capsule formation over implant etc. affect the performance of continuous measuring implantable biosensors^3,4^. Intermittent and continuous measuring sensors also suffer with problems of patient compliance due to associated pain during withdrawal of blood samples. An improved patient compliance can be obtained by reducing the invasive nature by implanting the sensor in the subcutaneous tissue and monitoring the levels externally over a prolonged period of time termed as the ‘Minimally invasive biosensors’. Glucowatch^®^ is an example of non-invasive biosensor based on electric current to extract glucose in a transdermal pad for measurement of glucose. An important limitation of Glucowatch is the requirement of continuous calibration tests using finger prick. Additionally other limitations include higher warm-up time, skin irritation and tough operating processes. Considering these limitations, optical methods of estimation seem to be more promising for developing minimally invasive systems^5^. Easy fabrication, low power requirement and use of NIR radiations for interference free estimation are other advantages of such minimally invasive biosensors. Implantable NIR fluorophore based biosensors were first described by McShane *et al*.^6^. Pyrene butyric acid, Diphenyl anthracene (DPA), Pyrene, Decacyclene, Ruthenium based fluorophores; Iridium trisbipyridine, Pt/Pd porphyrin and Europium are commonly used oxygen sensitive fluorophores which can be translated into glucose biosensors. Among them Pt/Pd porphyrin, Europium, Ruthenium based fluorophores are commonly used oxygen sensitive NIR fluorophores having emission wavelength greater than 600 nm^7^. Otto and Wolfbeis entrapped GOx in a thin film sol gel doped with oxygen sensitive ruthenium (II) (4, 7-diphenyl-1, 10-phenanthroline) 3-(dodecyl sulfate)_2_ (Ru(dpp)). The enzymatic reaction was monitored by the fluorescence of the oxygen-sensitive fluorophore, at high excitation wavelength of 465 nm and emission wavelength 610 nm^8^. Xu *et al*. studied a ratiometric estimation of fluorescence in polyacrylamide-based PEBBLEs (probes encapsulated by biologically localized embedding) i.e. GOx and an oxygen sensitive fluorescent dye, Ru[dpp(SO_3_Na)_2_]_3_)Cl_2_ (4,7-diphenyl-1,10- phenanthroline disulfonic acid disodium salt)^9^. For ratiometric analysis, another oxygen insensitive dye Oregon green 488 was also incorporated. Pt-Porhyrin is another class of oxygen sensitive fluorophore which has not been explored to a great extent for developing nanobiosensors for glucose. Due to its photo-stability, Pt-Porphyrin could find use as a dye for a stable biosensor. Pt/Pd-porphyrin acts as an oxygen sensitive fluorophore and has been traditionally encapsulated in hydrophobic carriers like silica based matrices e.g. organically modified silica (ORMOSILs), poly-fluorene derivatives, methacrylate derivatives, polystyrene etc^10–13^. Most of these carriers are not biodegradable and biocompatible which are important aspects in developing implantable biosensors.

The current research describes for the first time, encapsulation of Pt-Porphyrin in a hydrophobic, biodegradable and biocompatible polymer based nano/micro/hybrid particles which work as an oxygen detection system so that it can be used for developing a glucose biosensor. Oxygen sensitive nature of Pt-Porphyrin is utilized for detecting glucose catalytic reaction by GOx. In order to develop a biosensor matrix, a hybrid micro-carrier was formed where in Pt-Porphyrin loaded polymer nanoparticles along with enzyme GOx are co-encapsulated in a micro carrier. Alginate microspheres were chosen as hybrid bio-polymer carrier which can be implanted in subcutaneous tissue for continuous monitoring of glucose. The developed nanoparticle embedded microsphere containing Pt-Porphyrin and GOx were characterized using different techniques and tested for biosensor performance *in vitro*. The biosensors were also tested for biocompatibility using *in vitro* cell culture studies in L929 mouse fibroblast cell lines.

## Results and Discussion

### Preparation of Pt-Porphyrin loaded PEI-PLGA, PLGA, and PLA nanoparticles

Poly-lactide-co-glycolide (PLGA), Poly-lactic acid (PLA) are synthetic polymers which are known to be biodegradable. PLGA contains 50:50 lactide and glycolide monomer units. As a co-polymer lactide units provide the hydrophobicity and glycolide units provide hydrophilicity of the polymer. PLGA is easily dissolved in acetone and thereby can form organic phase in an emulsification process. For developing PLGA based nanoparticles, PLGA in acetone forms the organic phase and is poured in a surfactant solution (Tween) to form a fine emulsion. Acetone was evaporated in a rotavapor to form PLGA nanoparticles. Similarly, Polyethylene-imine-PLGA (PEI-PLGA) nanoparticles were also formed so that hydrophobicity of PLGA can be increased and a positively surface charged polymeric nanoparticle can be formed with an aim to increase the encapsulation of Pt-Porphyrin. PEI increases the hydrophobicity of the admixture polymer nanoparticles and also provides with a positive charge on the surface which can be used for electrostatic attachments. PEI was also chosen as it can be dissolved in acetone and is also known to stabilize proteins like enzymes and macromolecules. Pt-Porphyrin has a higher solubility in Tetrahydrofuran (THF) and Dichloromethane (DCM) with poor solubility in aqueous solvents. High volatility of solvents like DCM, ethyl acetate, and chloroform lead to formation aggregated particles systems of Pt-Porphyrin in the polymers investigated. Among the above solvents Pt-Porphyrin in acetone (0.02 mg/ml) produced nanoparticles of uniform shape and size when loaded in PLGA/PEI-PLGA/PLA nanoparticles using polyvinyl alcohol (PVA) (1% w/v) in an emulsification and solvent evaporation method. PVA serves to decrease surface tension and increase viscosity to stabilize the formed nanoparticles. PVA showed uniform formation of spherical nanoparticles in case of PEI-PLGA and PLA. However, PLGA nanoparticles were best formed with Tween 85^®^. Pt-Porphyrin has also been reported to be encapsulated with silica nanoparticles and organically modified silica nanoparticles by Prasad *et al*. and Koo *et. al*. for real time measurements of oxygen concentration^14,15^. Optical PEBBLE nanoparticles were developed for dissolved oxygen monitoring. Pt-Porphyrin and Pt-Porphyrin ketone have been encapsulated in silica nanoparticles, showing potential of encapsulation of both hydrophobic and hydrophilic moieties^15^. Porphyrin has been encapsulated in matrices like polystyrene, MTEOS, Octyl-TEOS, polyfluorenes etc.^16,17^. Most of these reports prominently describe presence of hydrophobicity in the polymers enabling encapsulation of Pt-Porphyrin. However most of the carriers used for Pt-Porphyrin encapsulation are either only biocompatible and are not degradable when used for *in vivo* purposes. Polymeric nanoparticles PLGA/PLA based materials can form important carriers as degradable and biocompatible encapsulation matrix for Pt-Porphyrin.

### Preparation of GOx-Pt-Porphyrin-PLA-alginate hybrid system (GPP-AM)

**Figure 1:**
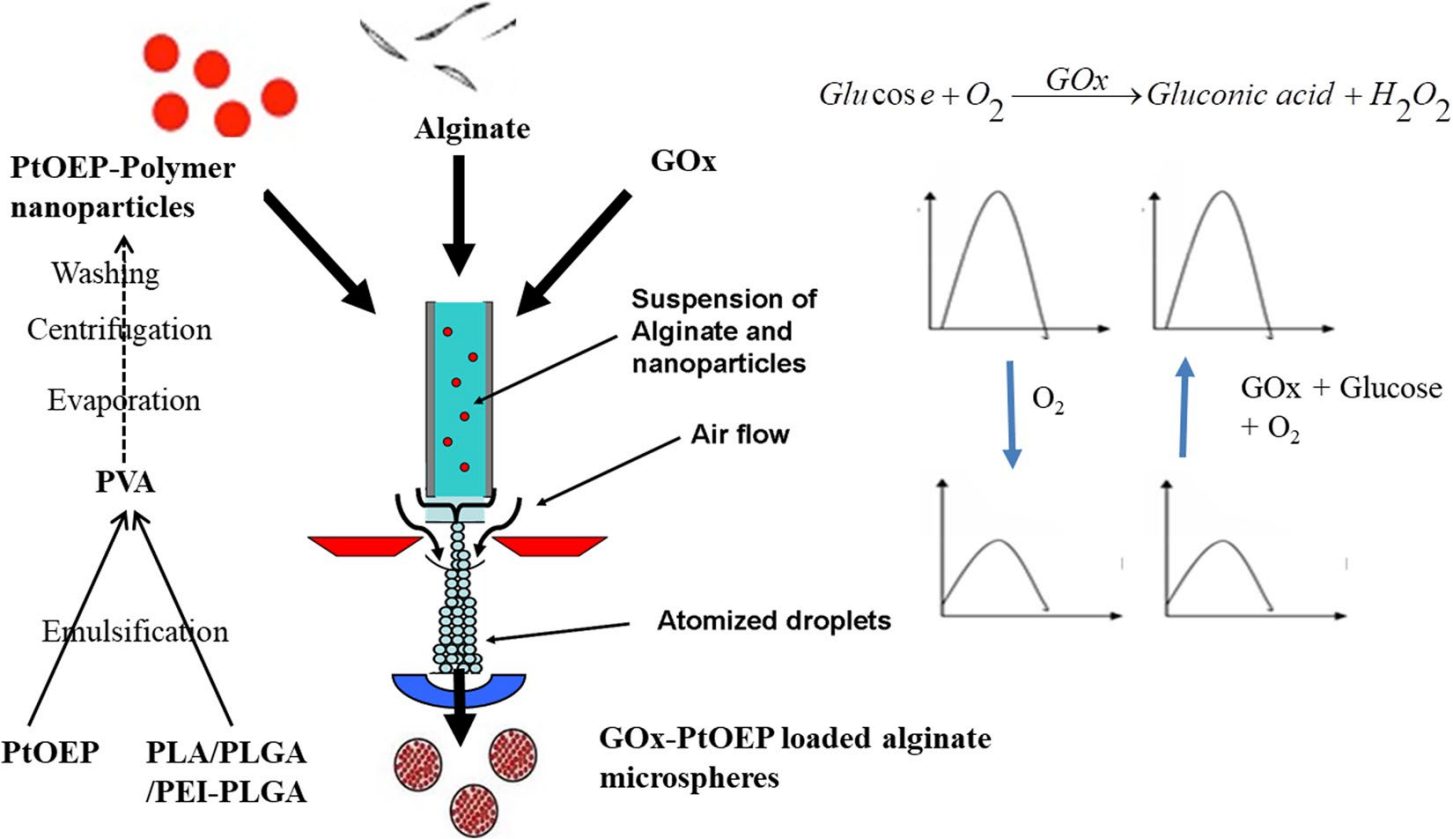
Schematic for optimization of production of nano-micro hybrid matrices of alginate for oxygen and glucose sensing.

GOx-Pt-Porphyrin-PLA-alginate hybrid system (GPP-AM) was developed using an adapted method from Joshi *et al*. to optimize influencing instrument related parameters like nozzle size (0.35 mm), pressure (450–500 mBar), flow rate (10–15 ml/hr), distance from nozzle to cross linking solution (5–10 cm). Optimization were carried out using alginate solution (2% w/v) sprayed in Calcium chloride (CaCl_2_)(250 mM) (Fig. 1)^18^. Porphyrin loaded nanoparticles were encapsulated in alginate microspheres and formulated based on the fact that properties of both nanoparticles and microspheres can be used for achieving advanced properties of biosensing. Nanoparticles can provide an improved sensitivity of detection due to greater surface area to volume ratio and microspheres can provide capability to encapsulate macromolecules in a stable and functional manner. Nanoparticles encapsulated using air driven atomization achieve a high encapsulation efficiency values due to nucleation and shear generation for forming hybrid matrices. Encapsulation of hydrophobic polymers like PLGA/PLA in a hydrophilic alginate makes these particles interesting carriers for multiple active components. Such hybrid matrices using gelatin, calcium carbonate, magnetic, per oxalate nanoparticles etc. have also been described to produce wide range of applications^18–21^. GOx is a macromolecule with molecular weight of 160 kDa is encapsulated in alginate micro particles in a single step process along with the Pt-Porphyrin loaded polymeric nanoparticles. The alginate matrix was selected due to its properties like biodegradation, mild gelation at room temperatures, and no additional chemicals used like oil, surfactants etc. The air driven atomization involves application of aerodynamic force where the polymeric solutions/suspensions while passing through an orifice nozzle (diameter = 0.35 mm) disrupts the droplets forming micron size particles. Such nanoparticles embedded matrices can also form the basis of implantable biosensors based on smart tattoo concept^6^ as the matrices can stay in subcutaneous tissue and cannot permeate through blood vessels. This is due to fact that micro-particles have lower migration and diffusion potential in comparison to nanoparticles when subcutaneously implanted.

### Characterization of nanoparticles and hybrid matrices

**Figure 2:**
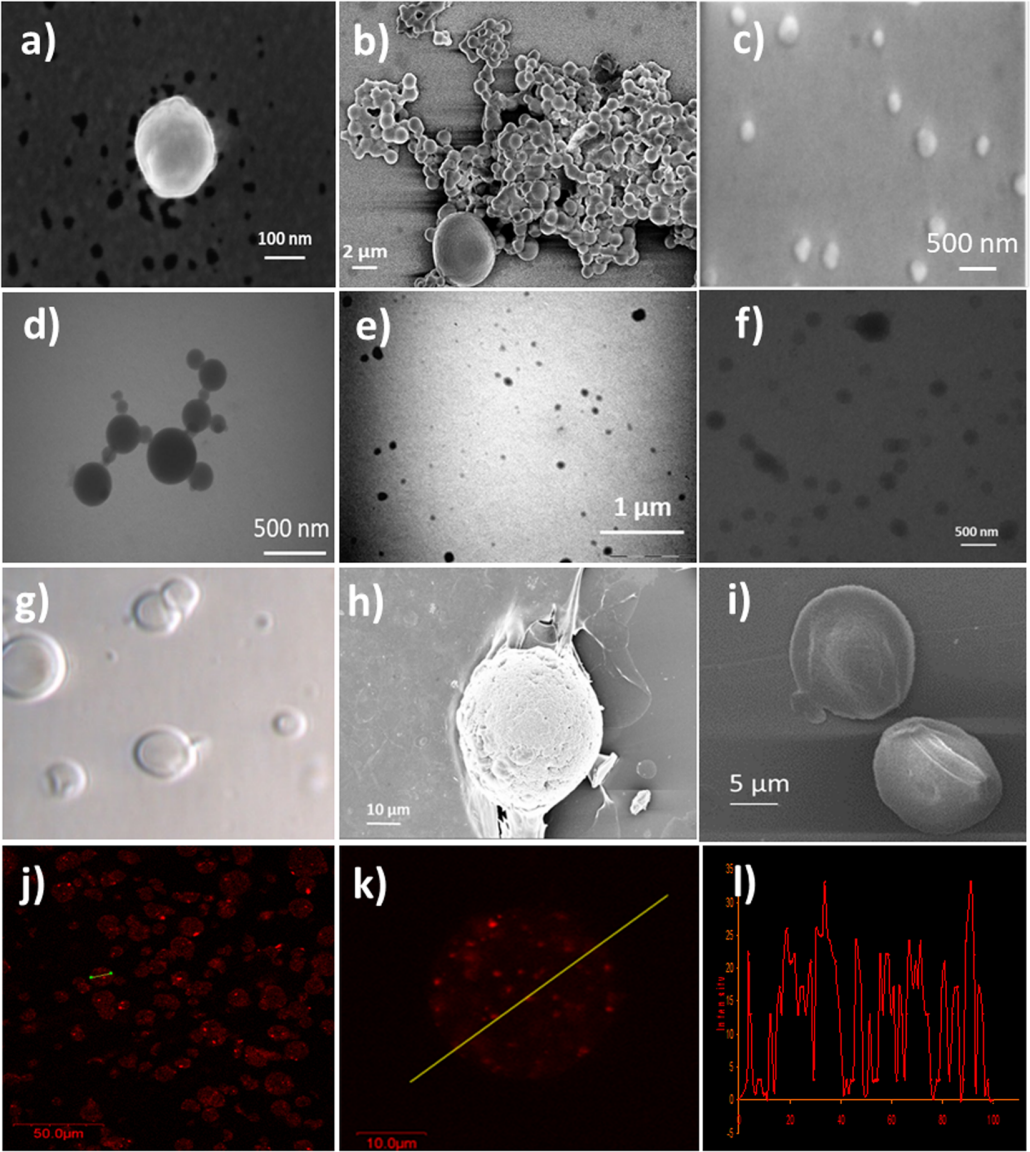
Characterization (**a**–**c**) SEM images of Pt-Porphyrin loaded PLGA, PEI-PLGA and PLA nanoparticles (**d**–**f**) TEM images of Pt-Porphyrin loaded PLGA, PEI-PLGA and PLA nanoparticles (**g**–**i**) Optical and SEM images of Pt-Porphyrin loaded hybrid alginate micro-carriers and (**j**–**l**) CLSM images of Pt-Porphyrin loaded hybrid alginate micro-carriers along with line scan imaging.

PLGA, PEI-PLGA and PLA nanoparticles formed from conventional emulsification solvent evaporation method were characterized using Dynamic light scattering (DLS). PLGA and PLGA-PEI nanoparticles showed a hydrodynamic diameter of 210 nm and 201 nm and polydispersity of 0.22 and 0.238, respectively. On the other hand PLA nanoparticles show a size of 256 nm and a polydispersity of 0.25. Upon encapsulation of Pt-Porphyrin the particle sizes in all these nanoparticles increase to 380, 350 and 450 nm, respectively. Transmission electron microscopy (TEM) images showed that the Pt-Porphyrin loaded nanoparticles formed spherical, non-aggregating particles (Fig. 2d–f). Higher Pt-Porphyrin loading however may lead to aggregation and deformed particles. The sizes were also confirmed using Atomic force microscopy (AFM) analysis (Fig. S1). Table 1 and Fig. 2 describes a comparison of sizes and zeta potential of different Pt-Porphyrin loaded nanoparticles of PEI-PLGA (350 nm), PLGA (210 nm) and PLA (450 nm). The zeta potential values of Pt-Porphyrin loaded PLGA, PEI-PLGA and PLA nanoparticles were found to be −10 mV, +15 mV and −15 mV, respectively which suggest that all the three nanoparticles are stable in suspension phase. Among them PLA nanoparticles possess highest stability indicated by a high negative value. PLA nanoparticles are more hydrophobic in nature in comparison to the PLGA and PEI-PLGA. This hydrophobicity is imparted due to an additional -CH_3_ moiety in lactide chains in comparison to glycolide chains. This additional hydrophobicity aids greater encapsulation of Pt-Porphyrin. In case of PLGA, PEI-PLGA nanoparticles the % loading of Pt-Porphyrin was found to be 55% and 61.8% for 0.02 mg/ml loading calculated using regression equation y = 86.155x + 0.0362 (Fig. S2a). The differences in encapsulation efficiencies in spite of similar emulsification procedures can be explained on the basis of differences in hydrophobicity. Hydrophobicity profiles are in the order of PLGA < PEI-PLGA < PLA. Greater hydrophobic nature of polymer supports higher encapsulation of Pt-Porphyrin dye. Therefore PLA nanoparticle show better encapsulation efficiency of Pt-Porphyrin for 0.02 mg/ml concentration of about 76%. GOx encapsulation as calculated from Bradford assay was found to be about 62% in hybrid matrix.

**Table 1:** Summary of sizing of plain nanoparticles, dye loaded nanoparticles, coated nanoparticles using dynamic light scattering and zeta potential values.

Type of nanoparticles	Size and polydispersity	Zeta potential
PLGA	210 nm (0.22)	−15 (0.9) mV
Pt-Porphyrin loaded PLGA nanoparticles	380 nm (0.28)	−10 (1.2)
PEI-PLGA	201 nm (0.238)	+12 (1.4) mV
Pt-Porphyrin loaded PEI-PLGA	350 nm (0.31)	+15 (2.2)
PLA nanoparticles	256 nm (0.25)	−20 (0.8) mV
Pt-Porphyrin loaded PLA nanoparticles	450 nm (0.31)	−15 (0.3)
GPP-AM particles	10 µm (±5 µm)	−25 mV

Summary of sizing of plain nanoparticles, dye loaded nanoparticles, coated nanoparticles using dynamic light scattering and zeta potential values.

The particle size of plain alginate microspheres was found to be 30 µm but when Pt-Porphyrin loaded PLA nanoparticles were loaded in alginate microparticles, the particle size was found to be 10 µm. Optical imaging and scanning electron microscopy (SEM) imaging were performed for microspheres, hybrid microspheres, respectively. The results indicate spherical particles with smooth surface and sometimes slightly rough surface when nanoparticles are loaded in alginate micro-carriers. The particle sizes were also confirmed with SEM imaging (Fig. 2a–c,g–i). SEM imaging describes that the particles formed using alginate and nanoparticles form porous structure which are necessary for analyte and product transport in the enzyme catalytic reaction for efficient biosensor function. Pt-Porphyrin loaded nanoparticles of PLGA and PEI-PLGA in alginate microspheres were also confirmed by confocal laser scanning microscopy (CLSM) (Fig. S1). Confocal image of Pt-Porphyrin loaded alginate micro-carriers showed uniformly dispersed discrete fluorescence from nanoparticles. The image clearly indicates successful formation of uniform Pt-Porphyrin loaded polymer nanoparticles loaded in alginate microspheres (Fig. 2j). This fact can also be supported by the line scan and the z scan (not shown) of the microspheres in Fig. 2k,l. Pt-Porphyrin loaded alginate micro-carriers were found to be formed with sizes smaller in comparison to plain alginate micro-carriers. The reason for this is the production of additional forces during atomization and formation of nuclei to build the droplets leading to development of small particles. Such results on capability to produce smaller particles sizes using nanoparticle embedded matrices have been described a report by Joshi *et al*.^18,20^. Table S1 describes effect of different instrumental parameters on particle size of PP-AM and GPP-AM particles.

### Oxygen Sensing Studies

Oxygen is a common quenching agent for many fluorophores. Pt-Porphyrin is one such fluorophore which shows a high quenching potential of fluorescence emission due to interaction with oxygen. The reduction in fluorescence emission can be described by Stern-Volmer equation which is given by

1 $$\frac{{I}_{0}}{I}={K}_{sv}[{O}_{2}]$$

**Figure 3:**
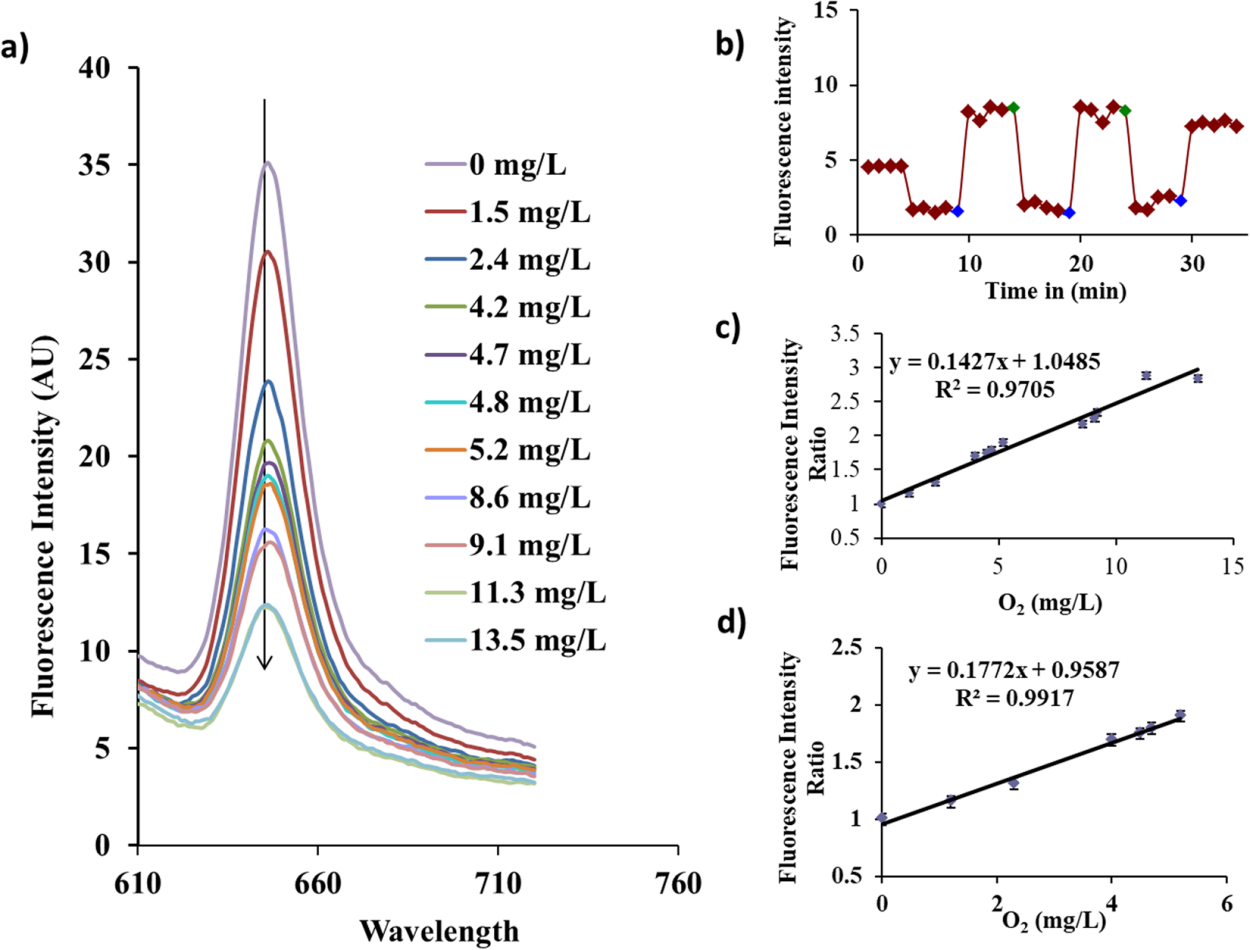
(**a**) Fluorescence emission overlay representing changes due to differences in oxygen concentration 0–14 mg/L of Pt-Porphyrin-PLA nanoparticles loaded hybrid alginate formulation with changing oxygen concentration. (**b**) Typical dynamic response curve for Pt-Porphyrin loaded PLA nanoparticles when bubbled with Nitrogen and Oxygen. (**c**,**d**) Calibration curves for hybrid microparticles in different ranges: 0–13 mM and 0–6 mM. Readings in the calibration curve were determined as mean values of n = 3 experiments and y error bars signify the standard deviation.

Where I_0_ and I are the fluorescence emissions intensities obtained without and with quencher. Ksv is the Stern-Volmer coefficient which is dependent on the matrix type and influences the diffusion of dissolved oxygen in the matrix and [O_2_] is the concentration of dissolved oxygen in the system. Oxygen sensing studies were carried out for Pt-Porphyrin loaded nanoparticles and Pt-Porphyrin-PLA-Alginate microspheres formed using different ratios of dye loaded nanoparticles. Figure 3a describes an overlay of fluorescence emission for Pt-Porphyrin-AM. A reversible response in terms of fluorescence emission intensities after consequent bubbling of nitrogen and oxygen cases was observed which clearly indicates oxygen sensitivity and capability to detect changes in oxygen levels (Fig. 3b). Linear regression profiles of oxygen detection show excellent regression coefficients in both 0–6 mM and 0–14 mM ranges (Fig. 3c,d). Table 2 indicates that dye loaded nanoparticles have good capability to detect changes in oxygen levels represented by a high Stern-Volmer coefficient (0.121) calculated from the slope of I_0_/I against concentration of oxygen. The response for nanoparticles was found to be following a logarithmic plot, however in 0–6 mM range, the sensing profile is linear in the range represented by a regression coefficient of 0.958. In case of implantable glucose sensors the oxygen changes occurring during catalytic reaction typically involve changes in the range of 0–2 mM which is completely covered by these Pt-Porphyrin loaded PLA nanoparticles^22^. PP-AM hybrid microspheres show reduced performance in terms of lower sensitivity in the linear range of 0–6 mM when compared against the Pt-Porphyrin loaded nanoparticles. This lowering of sensitivity occurs due to increase in the diffusion distance of oxygen molecules which have been exposed inside the microspheres. Considering the higher surface area to volume ratios of nanoparticles and a lower diffusion distance for nanoparticles the sensitivity as indicated by the Stern-Volmer coefficient was found to be higher in comparison to hybrid microspheres. The embedding of oxygen sensitive PP-AM nanoparticles inside the microspheres increases the diffusion distance of oxygen thereby reducing the sensitivity. However, increasing the concentration of nanoparticles in the alginate microspheres and hybrid microsphere concentration in the reaction mixture aids to improve the sensitivity of oxygen estimation.

**Table 2:** Comparison of oxygen sensing performance of Pt-Porphyrin based formulations like nanoparticles and hybrid micro-particles.

Sample		Equation	Range	R^2^	Ksv
Pt-Porphyrin-PLA nanoparticles		Y = 0.121x + 0.748	0–6 mM	0.958	0.121
Hybrid Microspheres	1:1	Y = 0.088x + 1.048	0–6 mM	0.948	0.088
	1:2	Y = 0.096x + 0.96	0–6 mM	0.953	0.096
	1:3	Y = 0.099x + 0.764	0–6 mM	0.970	0.099
	1:4	Y = 0.092x + 0.958	0–6 mM	0.926	0.092
	100 μl	Y = 0.099x + 0.764	0–6 mM	0.970	0.099
	200 μl	Y = 0.127x + 0.863	0–8 mM	0.972	0.127
	300 μl	Y = 0.142x + 1.048, Y = 0.177x + 0.958	0–13 mM, 0–6 mM	0.970 0.991	0.142 0.177

Owing to a higher solubility of oxygen in the hydrophobic matrices the PLA nanoparticles serve to be superior quenching systems in oxygen estimation with Pt-Porphyrin. This can also be related that if concentration of such Pt-Porphyrin-PLA nanoparticles is increased in a hydrophilic system the sensitivity can be increased. The result in this oxygen sensing study indicates that PLA nanoparticles are better carriers for dissolved oxygen in comparison to alginate due to its hydrophobic properties. However the effect can be improved by loading more amounts of dye loaded nanoparticles in the microspheres. This improvement can be seen as improvement in linearity indicative as regression coefficient. For 1: 1, 1: 2 and 1:3 formulations the regression coefficient increased from 0.948, 0.953 and 0.970. Further increase causes decrease in sensitivity of oxygen estimation owing to saturation of the effect.

A study on 100, 200 and 300 μl of PP-AM sensor suspension for oxygen sensing showed a similar effect as for different ratios. The linearity of the sensor was found to be improved in a greater range for e.g. 200 μl and 300 μl showed an improved range of 0–8 mM and 0–13 mM, respectively. A similar increase in Stern- Volmer coefficient was also seen. In case of 300 μl linear range of 0–6 mM showed a greater regression coefficient in comparison to all other formulations including nanoparticles. Highest Stern-Volmer coefficient was observed in case of 300 μl solution a value of 0.177 in comparison to 0.121 for nanoparticles. Therefore it can be concluded that the 300 μl of 1:3 formulation hybrid microsphere systems can give a better performance than nanoparticles in the range of 0–6 mM of oxygen concentration. Oxygen sensing based on PLA nanoparticles loaded with Pt-porphyrin with and without alginate microspheres have not been described in the literature till date. Previously, PLA nanoparticles have been described for oxygen sensing using Difluoroboron β-diketonate based materials encapsulated in PLA for tumor hypoxia detection using a portable inexpensive camera^23,24^.

### Glucose Sensing Studies using PEI-PLGA/PLA/PLGA nanoparticle and GOx-Pt-Porphyrin-PLA nanoparticle loaded alginate microspheres (GPP-AM)

**Figure 4:**
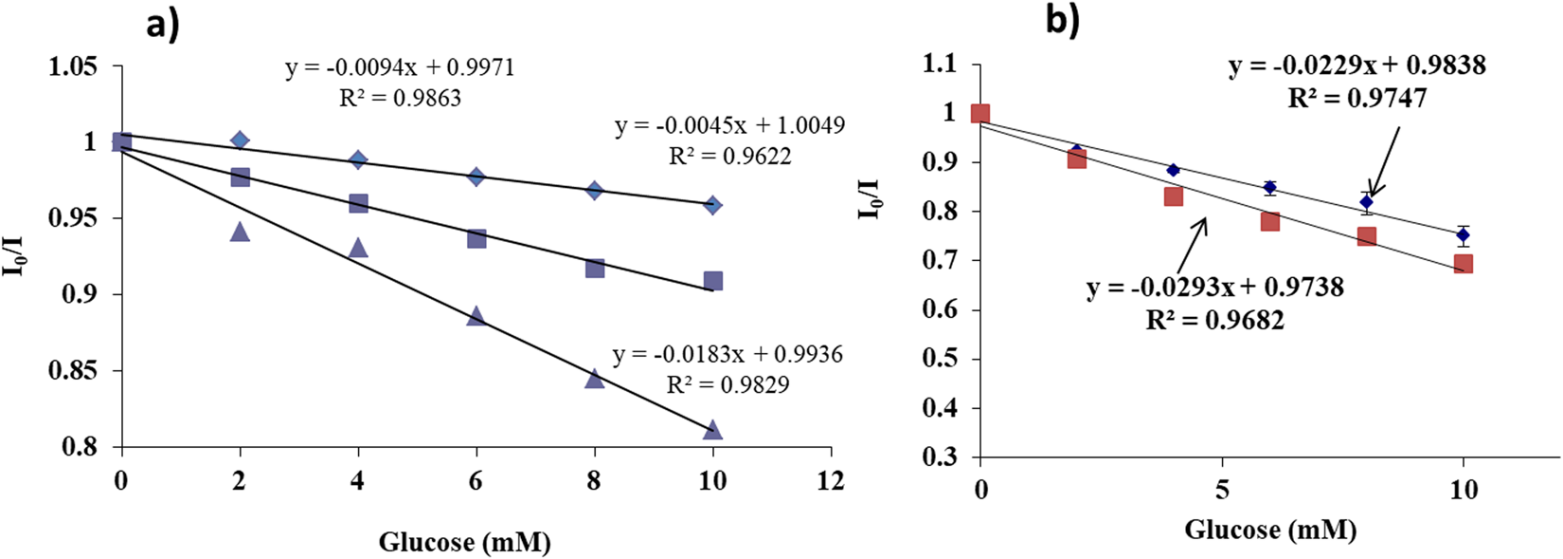
(**a**) Comparison of solution phase glucose sensing profiles of Pt-Porphyrin loaded polymeric nanoparticles (i) PLA nanoparticles (), (ii) PEI-PLGA nanoparticles () and (iii) PLGA nanoparticles () and (**b**) Glucose sensing behavior of GPP-AM hybrid particles in presence of simulated interstitial fluid () in comparison to deionized water ().

The main aim of this experiment was to understand the glucose sensing behavior and the sensitivities provided by polymeric nanoparticles produced from PEI-PLGA/PLA/PLGA. Suspension phase experiments of glucose and GOx (2 mg/ml) were carried out so that both of them are in solution and the Pt-Porphyrin nanoparticles are exposed to enzyme solution in an air tight cuvette. Upon the start of reaction, local oxygen depletion takes place leading to an increase in the fluorescence intensity of Pt-Porphyrin. This increase in intensity is recorded in the form of a Stern-Volmer plot, where in I_0_/I was calculated and plotted against the glucose concentrations. Statistically significant differences in sensitivities of polymeric nanoparticles were observed when different polymeric carriers were tested. The results suggest that the Ksv value for PLGA, PEI-PLGA and PLA nanoparticles were found to be 0.0094, 0.0045, and 0.0183/mM of glucose which indicates that the diffusion pattern for oxygen analyte produced from the reaction was better in case of PLA nanoparticles in comparison to PLGA and PEI-PLGA nanoparticles (Fig. 4a). Results also indicate that the sensitivity value for PLA nanoparticle was found to be 1.9% change in emission intensity ratio/mM of glucose in comparison to 0.413% change in emission intensity ratio/mM glucose and 0.9% change in emission intensity ratio/mM glucose in case of PLGA and PEI-PLGA nanoparticles, respectively (Fig. 4a). Statistically significant differences in sensitivity values p < 0.05 values were observed in the performance of different nanoparticles for suspension phase glucose sensing. Pt-Porphyrin loaded in PLA nanoparticles was found to be superior in comparison to PLGA and PEI-PLGA nanoparticles. Based on the values of high encapsulation efficiency and suspension phase sensitivity of PLA nanoparticles they were used for developing GPP-AM particles.

Glucose oxidase in solution interacts with glucose to form gluconic acid and hydrogen peroxide by consuming oxygen. The depletion of oxygen levels can be measured using GPP-AM due to encapsulated Pt-Porphyrin. An increase in fluorescence intensity in response to addition of glucose (0–50 mM) was observed when fluorescence scans were captured using a λex of 530 nm. The response was determined to be linear within 0–10 mM with a Stern-volmer coefficient of 0.0293, after which the response was found to be saturated with 2 mg/ml GOx enzyme (Fig. 4b). The sensitivity of glucose determination was found to be 3.73% change in emission intensity ratio/mM of glucose when the biosensing was performed in deionized water. Since the proposed sensor is of implantable nature which can be injected subcutaneously, simulated interstitial fluid was chosen to investigate possible interferences. Glucose sensing performance was evaluated using a simulated interstitial fluid which does not contain glucose developed using previous reports^25^. The study indicated that there was no significant difference in both the linearity and range of detection when compared against the biosensing performance obtained in deionized water. The slope of linear range indicates the rate of reaction of glucose and corresponding changes in the concentration of oxygen. The increase in sensitivity of GPP-AM in comparison to suspension phase sensing of GOx and Pt-porphyrin loaded PLA nanoparticles can be achieved by using high concentration of Pt-Porphyrin in PLA nanoparticles. These nanoparticles along with GOx in the microenvironment of alginate carriers allows for poor quenching of Pt-porphyrin fluorescence due to greater utilization of oxygen by GOx catalytic reaction.

### Validation of Glucose sensor performance

Glucose sensing performance of GPP-AM was evaluated by studying several validation parameters like Linearity, Regression coefficient, Accuracy, precision, reproducibility, response time, stability and limit of detection (LOD). Linear regression analysis showed a good regression coefficient of 0.968 within 0–10 mM range with an equation of y = −0.0293x + 0.9738 using a very low sample volume (50 µl). When the stability of the formulation was evaluated, sensor response was stable for 15 days upon storage at 4 °C. Precision studies showed that the standard deviation of glucose measurement for 8 mM glucose concentration was found to be about 2.09% RSD (n = 6). The accuracy studies determined as % recovery values lie in the range of 98–115% for concentrations 4, 6, and 8 mM. The intra-day and inter-day standard deviation of glucose response curve was found to be 1.8% RSD and 3.6% RSD, respectively. The response time of glucose detection using GPP-AM, when tested for 10 mM of glucose was found to be about 4 minutes (Fig. S3). LOD determined from the calibration curve were found to be 1.5 mM. Fig. S2 describes the reversibility profile for different glucose concentrations in 0–10 mM.

### Enzyme activity and leaching studies

**Figure 5:**
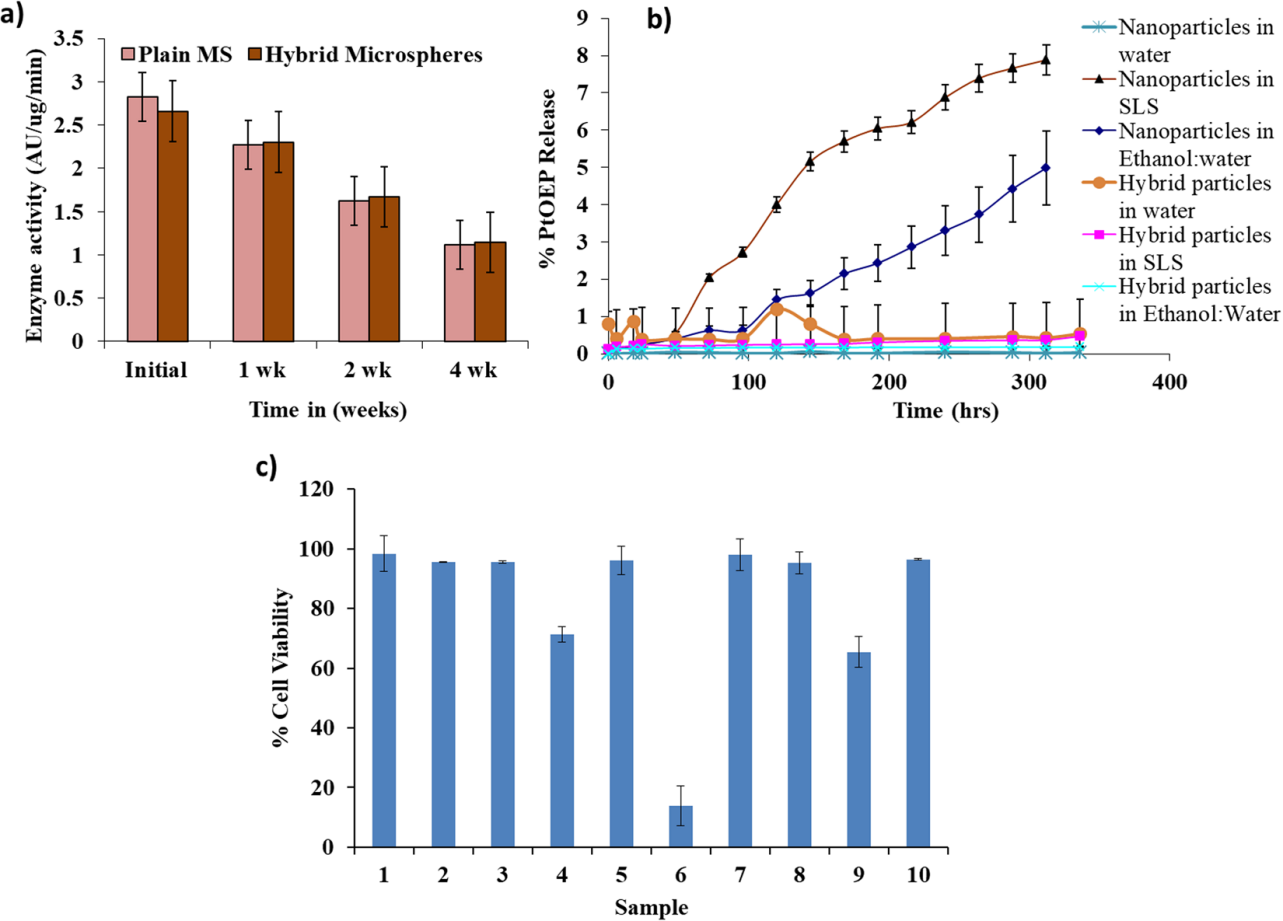
(**a**) Enzyme activity studies of GOx in GOX-Pt-Porphyrin-Alginate hybrid microspheres in comparison to plain alginate microspheres. (**b**) Leaching profiles of Pt-Porphyrin from PLA nanoparticles and hybrid matrices in different solvents like water, SLS and Ethanol: water mixture. (**c**) % cell viability on exposure to nanoparticles and hybrid microparticles using L929 mouse fibroblast cell line with test groups as DMSO (1), PLA nanoparticles (2), PLGA nanoparticles (3), PEI-PLGA nanoparticles (4), Alginate microspheres (5), Pt-Porphyrin in DMSO (6), Pt-Porphyrin in PLA (7), Pt-Porphyrin in PLGA (8), Pt-Porphyrin in PEI-PLGA (9), Pt-Porphyrin based hybrid microspheres (10).

The effect of co-encapsulation of GOx with Pt-Porphyrin loaded nanoparticles was tested using activity testing of GOx using *o*-dianisidine assay for GOx loaded alginate microspheres and GOx-Pt-Porphyrin-PLA co-encapsulated hybrid microspheres. The comparison of 10 min activity profile were evaluated to indicate that enzyme activity at zero time for plain alginate microspheres and hybrid microparticles was found to be 2.82 and 2.65 AU/µg/min (Fig. 5a). Statistically significant differences were observed when enzyme activity was compared at the end of 4 weeks in comparison to initial enzyme activity. After 7 days the drop in enzyme activity for the above formulations was 19.6% and 13.3% in comparison to the activity at zero time. At the end of 28 days a 60% and 56.8% drop in enzyme activity occurs. Such a drop in activity may occur due to leaching of enzymes and inactivation during storage over a period of time. The result also clearly justifies the sensor activity values to be maintained for about 15 days. Leaching of oxygen sensitive dye Pt-Porphyrin from PLA nanoparticles and hybrid particles was evaluated in different media like de-ionized water, 1% Sodium lauryl sulphate (SLS) and 1:1 ethanol water mixture. The leaching profiles suggest that less than 7% of the dye is released in deionized water from both dye loaded nanoparticles and hybrid micro-particles over a period of 15 days (Fig. 5b). The leaching profile of Pt-Porphyrin was found to be negligible suggesting the sensing chemistry can remain stable for a long duration of time.

### *In vitro* cytotoxicity testing

*In vitro* cytotoxicity testing was performed using a sulforhodamine B (SRB) assay which is based on interaction of the dye with proteins present in cells which are fixed to culture plates using trichloroacetic acid. The purpose of this study was to assess the *in vitro* cytocompatibility of diverse constituents of the biosensor viz. fluorophores, enzyme and nano/nano-microparticles using L929 cell line^26^. Different aspects of these cells like adhesion, proliferation, morphology were studied. The results show that the cells were able to have properties like adhesion, morphology, growth and viability of cells upon exposure to GOx-Pt-Porphyrin-PLA-Alginate hybrid particles. The Percent viability for Pt-Porphyrin in Dimethyl sulphoxide (DMSO) was found to be around 14% in comparison to cell viability in DMSO control. The presence of surfactant along with nanoparticles of polymers significantly reduces the cell viability as they might affect the cell membrane integrity. Thus complete removal of surfactant is essential from nanoparticles. When Pt-Porphyrin is encapsulated in PLA/PLGA/PEI-PLGA nanoparticles the viability of cells was found to be higher relative to Pt-Porphyrin in DMSO or unwashed Pt-Porphyrin-polymer nanoparticles. PLA/PLGA nanoparticles and alginate microspheres have been previously studied and found to be biocompatible and biodegradable and thus showing a high value for cell viability. PEI-PLGA nanoparticles showed slight toxicity to cells with about 70% cell viability (Fig. 5c). All the hybrid formulations of polymer nanoparticles in alginate microspheres show a good biocompatibility. This may be due to reduced contact of materials like PEI or dye with cells in a 48 hours duration of the study. This can be further correlated to the leaching studies that since very low amount of dye leaches out from the matrices, the cell viability is highly improved. This proves that encapsulation of Pt-Porphyrin in a polymeric matrix will reduce the exposure to cells so that the biocompatibility is improved. Such hybrid microspheres have also been investigated previously for biocompatibility and biosensor performance and results obtained by these reports are also in line with these results^14,19,21,25^.

## Experimental Section

### Materials

Polyethylene imine (PEI), Polylactide-co-glycolide (PLGA), Polylactic acid (PLA), Alginate (Low viscosity, 2%), Platinum octaethyl porphyrin (Pt-Porphyrin), *o*-dianisidine, Horseradish Peroxidase (HRP), Glucose, Glucose Oxidase (Type VII obtained from *Aspergillus Niger*) (GOx), Poly-vinyl alcohol (PVA) and Glucose were procured from Sigma Aldrich, India. Calcium chloride, Dicholoromethane, Acetone, Ethanol, Tween 85, Dimethyl sulphoxide (DMSO) and Sodium lauryl suphate (SLS) were procured from Merck Mumbai, India. All chemicals were reagent grade and were used as received. L929 (Mouse fibroblast) cell line was procured from National Centre for Cell Science (NCCS), Pune, India. DMEM (Dulbecco’s Medium Eagle Medium, Sigma, USA), FBS (Fetal Bovine Serum, Sigma, USA), trypsin-EDTA solution, trichloroacetic acid (TCA, Loba Chemie, India) and Sulphorhodamine B (SRB), Sigma-Aldrich Chemie, USA) were procurd to use them for cell culture experiments.

### Preparation of Pt-Porphyrin loaded PEI-PLGA, PLGA and PLA nanoparticles

Nanoparticles of PEI-PLGA, PLGA and PLA were prepared by emulsification-solvent-evaporation technique. Briefly, a solution of PLGA (50:50) in dichloromethane (10% w/v) and PEI solution in acetone (0.1% w/v) was stirred for 30 min maintaining PEI to PLGA weight ratios of 29:1. PEI-PLGA solution and Pt-Porphyrin in acetone (0.002%w/v) as organic phase were mixed with Tween^®^-85 (1% w/v), PVA (5% w/v, 10 ml), followed by sonication and evaporation of solvents using a rotavapor^27^. In case of PLGA nanoparticles, Pt-Porphyrin solution (0.002% w/v, 15 ml) and PLGA (75 mg) in acetone were mixed as organic phase water/alcohol mixture (1:1 v/v), containing 0.5% (w/v) of Tween^®^ 85. Similarly, Pt-Porphyrin (0.002% w/v) and PLA (5 mg/ml) were dissolved in acetone and poured in PVA (1%w/w). The nanoparticles were washed and concentrated by centrifugation at 25000 g for 30 min thrice using de-ionized water.

### Preparation of Pt-Porphyrin-PLA alginate hybrid microcarriers (PP-AM) and GOx-Pt-Porphyrin-PLA-alginate hybrid system (GPP-AM)

Pt-Porphyrin loaded PLA nanoparticles were suspended in alginate (2%w/w) solution and atomized to form a nanoparticle-microsphere hybrid system using a modified air driven atomization method as described by Chaudhari *et al*. and Joshi *et al*.^18–21^. Briefly, different proportions of alginate solution and Pt-Porphyrin loaded nanoparticles like 1:1, 1:2, 1:3 and 1:4 were sprayed using optimized influencing parameters (like flow rate 15 ml/hour, pressure 500 mbar, nozzle size 0.35 mm and distance 10 cm). The sprayed droplets were ionically crosslinked in CaCl_2_ (250 mM). The hybrid product was purified with deionized water to eliminate excess CaCl_2_ and stored between 2–8 °C^20,21^. This hybrid micro-carrier formulation would serve as an oxygen sensor (PP-AM). GOx encapsulation (2% w/v) in PP-AM was carried by mixing GOx in alginate using this process to develop oxygen sensing based Glucose biosensor (GPP-AM).

### Characterization of nanoparticles and hybrid matrices

Particle size and distribution of Pt-Porphyrin loaded polymeric nanoparticles were performed using DLS analysis, SEM and TEM imaging. DLS measurement of unloaded, Pt-Porphyrin loaded nanoparticles and electrophoretic mobility determination of nanoparticles were calculated using Brookhaven instruments using a correlation curve and Smoluchowski equation, respectively. BIC Zeta-PALS zeta potential analyzing software was used to analyze the surface charge. Dye loaded polymeric nanoparticles and GPP-AM hybrid particles were imaged using scanning electron microscopy (SEM) (Hitachi S-3400, Japan) by placing samples over carbon tape with a gold sputtered coating for examination at 150–3000X. TEM imaging was carried out to understand the morphological properties of nanoparticles using Transmission Electron Microscope (TEM) (PHILIPS, CM200). Samples were examined at ~200 kV using TEM in a carbon coated-copper grid after drying for an hour using a drop of nano-suspension. AFM imaging (VICO Nanoscope IV) was also performed on polymeric nanoparticles to understand the surface morphology using tapping mode. Optical microscopy was used for imaging of alginate hybrid nano-microparticles at different magnifications (10X and 40X) using a Nikon optical microscope. Encapsulation of GOx was determined by Bradford reagent by analyzing the supernatants. Encapsulation efficiency of Pt-Porphyrin was determined by a direct method by dissolving and extracting Pt-Porphyrin dye in DCM and THF. Pt-Porphyrin dye was quantified using UV spectroscopy a previously developed calibration curve in THF in the range of 0–6 μg/ml at 530 nm.

### Oxygen Sensing Studies

Pt-Porphyrin loaded PLA nanoparticles and different proportions of Pt-Porphyrin-PLA encapsulated nanoparticles in calcium alginate microparticles *viz*. 1:1, 1:2, 1:3, 1:4 were evaluated for oxygen biosensing ability in a protocol similar to Prasad *et al*.^14,18,19^. Fluorescence spectroscopy (with a 150 W Xenon lamp) was used with excitation wavelength of 530 nm and emission wavelength of 645 nm while controlling O_2_ bubbling in the samples to determine the oxygen sensitivity. The oxygen concentration was measured using an Orion DO meter (Thermo-Fischer Scientific, India). A Stern-Volmer plot was used to evaluate all the samples for oxygen diffusion coefficient determined by bubbling oxygen standards (in the range 0.77–16.0 mM). Pt-Porphyrin loaded nanoparticles/microparticles were exposed to different oxygen concentrations (produced by controlled bubbling of oxygen and nitrogen gasses at controlled flow and temperature) and fluorescence emission intensities captured.

### Glucose Sensing Studies using PEI-PLGA/PLA/PLGA/nanoparticle and GOx-Pt-Porphyrin-PLA-alginate hybrid system (GPP-AM)

Pt-Porphyrin loaded nanoparticles (PEI-PLGA/PLA/PLGA) were washed to remove surfactant and suspended in double distilled water. Pt-Porphyrin loaded nanoparticle suspension (100 µl) was mixed with GOx (2 mg/ml) and 100 µl glucose solution (0–50 mM) and volume finally made up to 1 ml. The fluorescence response (λ_ex_ = 530 nm and λ_em_ = 645 nm) was measured at every 30 seconds for a period of 10 minutes. Saturation intensity was measured when the intensity of fluorescence gets stabilized due to oxygen consumption. Similar, protocol was employed for testing the glucose biosensor performance in presence of simulated interstitial fluid which was developed without glucose as per components and concentrations adapted from Chaudhary *et al*. in order to investigate interference from normal biological components^25^. An intensity ratio was plotted with respect to concentrations of glucose. GPP-AM particles were exposed to variable levels of glucose (0–50 mM). The changes in fluorescence emission intensity at 645 nm with an excitation wavelength of 530 nm on exposure to glucose were monitored at the intervals of 30 seconds for 10–15 minutes. The saturation intensities were used for calculating the intensity ratios which were plotted against the concentrations to determine Stern-Volmer coefficient.

### Validation of sensor performance

The sensor performance was validated as per the ICH Q2A (International conference of harmonization tripartite) guidelines for analytical methods^28^. Several parameters like response time, accuracy, precision specificity, linearity, range, repeatability, reproducibility, limit of detection (LOD), reversibility of the sensor were calculated and evaluated. Response time was calculated from intensity ratios based on the achieving saturation intensity during an increase in Pt-Porphyrin emission intensity. Accuracy was represented by % recovery of the sensor calibration curve which was determined by independently examining three different concentrations in triplicate. A % recovery profile against theoretical concentrations was plotted and % RSD determined. Linearity and range were determined on data points of linear regression curve. Reversibility was tested using different samples in the calibration curve range on same set of sensor particles measurements carried out by washing the particles after each measurement. LOD of biosensor was determined from the same calibration curve using mathematical equation.

### Stability studies

Enzyme activity of GOx was evaluated by a colorimetric method having a principle based of the redox reaction of *o*-Dianisidine through a peroxidase system. The assay mixture consists of 0.21 mM in 0.01 M PBS buffer pH 7.4 *o*-dianisidine solution (2.4 mL), 100 mg/mL in 0.01 M PBS buffer (0.5 mL) of β-D (+)-glucose solution and 60 units/mL peroxidase solution (0.1 ml) in deionized water. 100 µl of GOx solution/GOx-loaded hybrid alginate microspheres dispersed in DI water were added to the assay mixture and the absorbance measured at 500 nm. The activity was monitored over 15 min duration to understand the catalytic profile. The enzyme activity was monitored over a period of 4 weeks in triplicate every week to obtain accurate results. The stabilization of dye inside the matrix was tested by studying the leaching of Pt-Porphyrin from Pt-Porphyrin loaded nanoparticles and Pt-Porphyrin loaded hybrid matrix. The leaching study was carried out by dialysis membrane method for about a month, wherein the samples were allowed to release the dye in release medium like water, 1% SLS, ethanolic water (50:50). The release medium was collected at specified intervals and analyzed using fluorescence spectroscopy at 645 nm for presence of any dye in release medium.

### *In vitro* cytotoxicity testing

*In vitro* biocompatibility studies of different samples were done using L929 (Mouse fibroblast) cell lines. The cells were raised and maintained in DMEM medium augmented with 10% FBS along with 1% antimycotic antibiotic solutions (37 °C, 5% CO_2_ and saturated humid environment). Adherent cells in 25 cm^2^ tissue culture flask were trypsinized using trypsin-EDTA solution and centrifuged at 1000 g for 10 minutes. The cell pellet was re-suspended in fresh media and added in each well of a 96 well plate (1 × 10^4^ cells). The cells were incubated in a CO_2_ incubator and after 24 hrs, the samples were added in the culture medium. After 48 hrs, Sulphorhodamine B (SRB) assay was conducted using a protocol adapted from Skehan *et al*. and Vichai *et al*.^26,29^. Briefly, old media with the samples were discarded and 200 µl complete media was added. 50 μl of ice-cold 50% trichloroacetic acid was added slowly to media with incubation for 1 hour at 4 °C. The plates were washed for 5 times and dried using air. 100 μl of 0.4% SRB was dissolved in 1% acetic acid and added to the fixed cells incubated for 20 minutes at room temperature. The excess SRB was washed away using 1% acetic acid. The plates were dried using 100 μl of 10 mM Tris base and incubated for 20 min to dissolve away the unbound dye. Absorbance of each well was taken in a plate reader at 560 nm. The readings were used to calculate the percentage viability by the relation, As/Ac x 100, where As/Ac is absorbance of sample and control, respectively.

### Statistical Analysis

Data are represented as Mean ± Standard deviation of triplicate (n = 3) measurement for all sensing experiments. Statistical comparisons and differences among groups were analyzed using student t-test and ANOVA.

## Conclusions

Novel biocompatible and biodegradable carriers for Pt-Porphyrin fluorophore have been described which can function as a NIR based oxygen sensitive fluorophore. Pt-porphyrin encapsulated PLA nanoparticles developed using emulsification-solvent evaporation provides promising results on oxygen sensing with a wide linear range and good sensitivity. Glucose oxidase based co-immobilized matrix along with Pt-porphyrin loaded PLA nanoparticles yielded a glucose biosensor which is developed using an atomization method. The developed glucose sensors were tested in simulated interstitial fluid for biosensing performance were able to detect glucose without any interference when compared against the biosensing performance obtained using deionized water. Pt-Porphyrin based oxygen sensing for glucose detection can bring advance features like near infra-red (NIR) emission causing an interference free estimation in implantable biosensors. The findings of current research suggest that oxygen detection and glucose detection is possible for physiological levels of oxygen and glucose with a short response time. The results show that proposed biosensor has great potential to be translated into a NIR emission based implantable glucose biosensor for continuous glucose measurement and monitoring.

## Supplementary information

Supplementary info
