# Supplementary material for: Fluorescent Biocompatible Platinum-Porphyrin–Doped Polymeric Hybrid Particles for Oxygen and Glucose Biosensing
Source: Sci Rep. 2019 Mar 22;9:5029. doi: 10.1038/s41598-019-41326-7 (PMC6430792; doi:10.1038/s41598-019-41326-7)
Supplement: Supplementary file 1 — Supplementary info [file 41598_2019_41326_MOESM1_ESM.docx]

**Fluorescent Biocompatible Platinum-Porphyrin–Doped Polymeric Hybrid Particles for Oxygen and Glucose Biosensing**

Gaurav Pandey ^1^ **^, ζ^**, Rashmi Chaudhari ^2^**^, ζ^**, Bhavana Joshi ^1,^ **^ζ^**, Sandeep Choudhary^1^ , Jaspreet Kaur^1^, Abhijeet Joshi **^1, *^**

**Supplementary Information**

**A)**

**
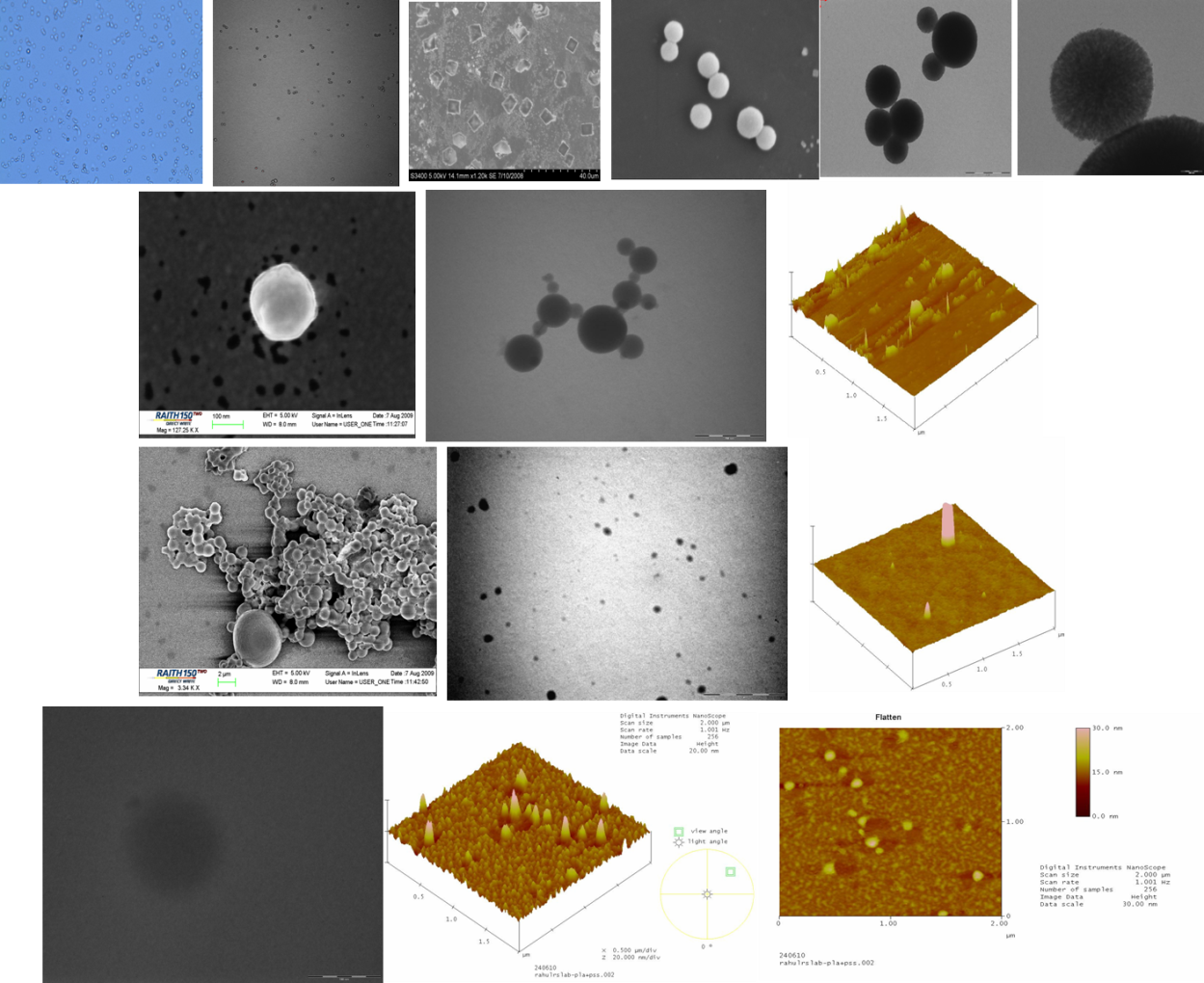

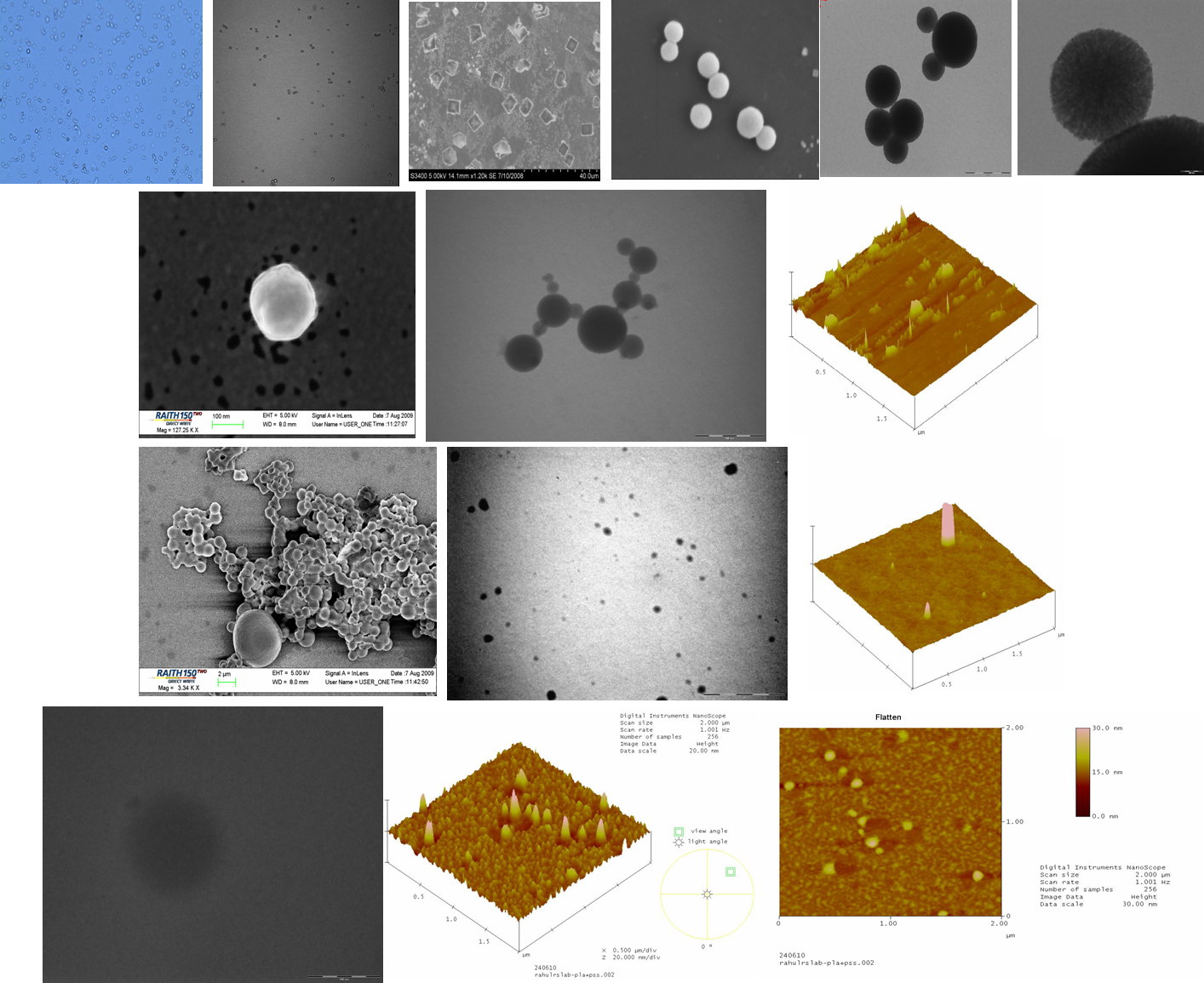

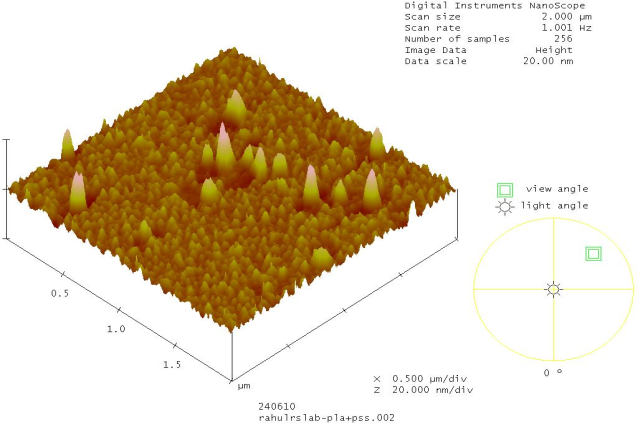
**





**B)**





**Figure S1: AFM images of PLGA nanoparticles, PEI-PLGA nanoparticles and PLA nanoparticles and CLSM images of PEI-PLGA nanoparticles embedded in alginate microspheres (A) and CLSM images of PLGA nanoparticles embedded alginate microspheres (B)**

a)

b)

**Fig S2: a) Calibration curve for Pt-Porphyrin for quantifying the encapsulation efficiency. b) Reversibility of response of Pt-Porphyrin when exposed to different concentrations of oxygen ranging from 0-10 mM**

**Fig S3. Response time curves for Glucose sensing using GPP-AM using a concentration of 6 mM Glucose, (Y error bars represent standard deviation of intensities of triplicate measurement**

**Table S1. Effect of Pressure and flow rate of droplet generator for production of PP-AM and GPP-AM carriers**

| **Pressure**  **(mbar)** | **Flow rate**  **(ml/hr)** | **Concentration nanoparticles: alginate** | **Size of Hybrid microparticles (µm)** |
| --- | --- | --- | --- |
| 70 (5) | 20 (2) | - | 60 (10) |
| 60 (5) | 10 (2) | 25 % w/v | 60 (10) |
| 120 (5) | 10 (2) | 25% w/v | 25 (7) |
| 300 (5) | 15 (2) | 75 % w/v | 15 (5) |
| 400 (5) | 15 (2) | 75 % w/v | 8 (5) |

The standard sample parameters used were concentration of alginate (2%w/v), Concentration of CaCl_2_ (250 mM), Nozzle size (0.35 mm) and distance for crosslinking (10 cm). Values in parenthesis represent the standard deviation; All the experiments were conducted for triplicate
